# Supplementary figures and images for: The complete chloroplast genomes of three Betulaceae species: implications for molecular phylogeny and historical biogeography
Source: PeerJ. 2019 Jan 25;7:e6320. doi: 10.7717/peerj.6320 (PMC6348958; doi:10.7717/peerj.6320)

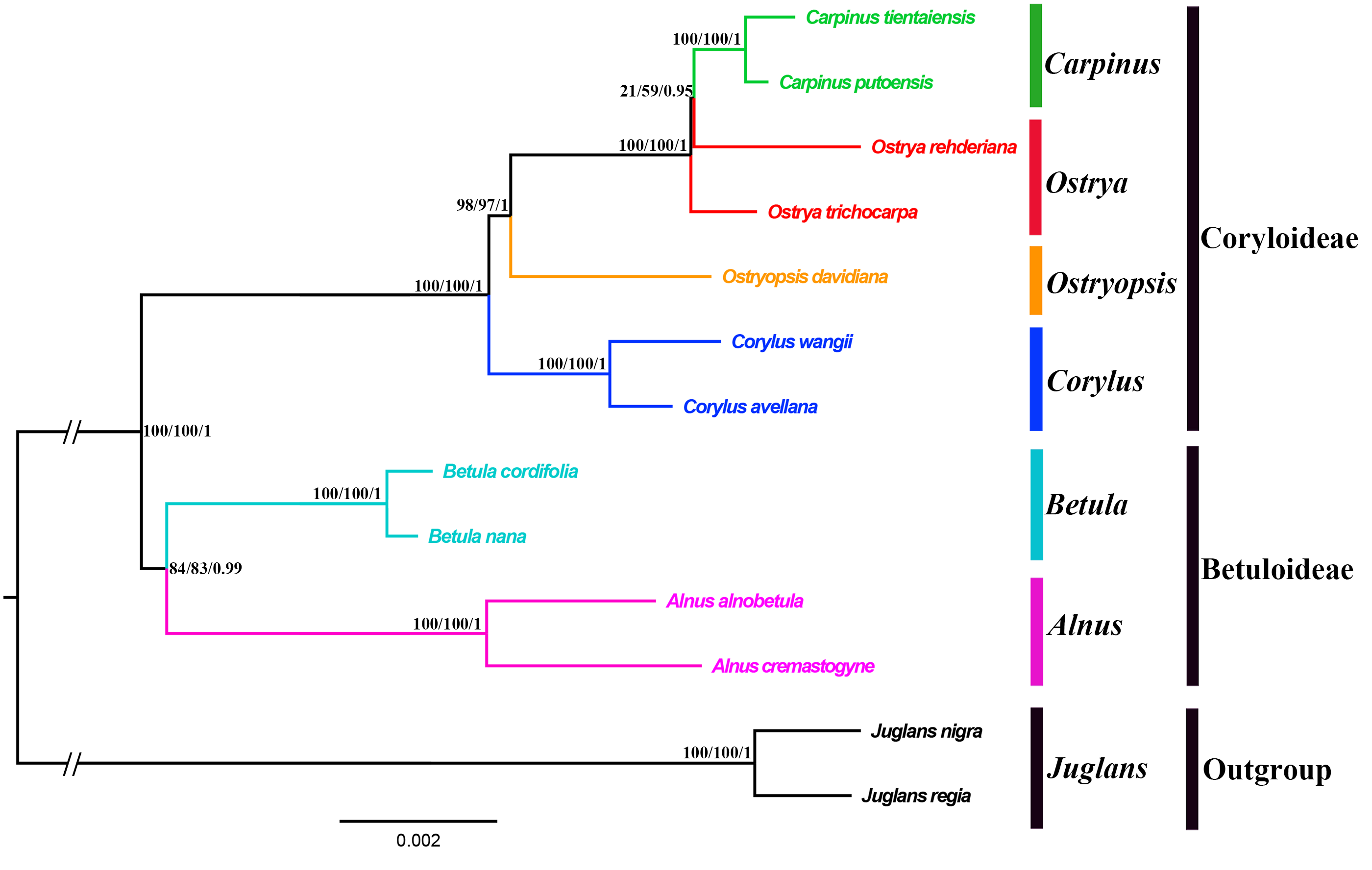

Supplement: Figure S1 — Support values of ML-SH-Alrt, ML-UFBoot and BI-PP are successively listed above the branches (SH-aLRT/UFBoot /PP). [file peerj-07-6320-s001.png]
